# Supplementary material for: Chemoenzymatic synthesis of 3-ethyl-2,5-dimethylpyrazine by L-threonine 3-dehydrogenase and 2-amino-3-ketobutyrate CoA ligase/L-threonine aldolase
Source: Commun Chem. 2021 Jul 16;4:108. doi: 10.1038/s42004-021-00545-8 (PMC9814548; doi:10.1038/s42004-021-00545-8)
Supplement: Supplementary file 1 — Supplementary Information [file 42004_2021_545_MOESM1_ESM.pdf]

Supplementary Information

Chemoenzymatic synthesis of 3-ethyl-2,5-dimethylpyrazine by L-threonine 3-dehydrogenase and 2-amino-3-ketobutyrate CoA ligase/L-threonine aldolase

Tomoharu Motoyama[1], Shogo Nakano[1,2,*], Fumihito Hasebe[1], Ryo Miyata[1], Shigenori Kumazawa[1], Noriyuki Miyoshi[1] and Sohei Ito[1,*]

[1] Graduate School of Integrated Pharmaceutical and Nutritional Sciences, University of Shizuoka, Shizuoka, Japan

[2] PREST, Japan Science and Technology Agency

*To whom correspondence may be addressed: Graduate School of Integrated Pharmaceutical and Nutritional Sciences, University of Shizuoka, Shizuoka, Japan, Tel.: +81-54-264-5528 (Ext.); Fax: +81-54-264-5528, E-mail: snakano@u-shizuoka-ken.ac.jp

ORCID ID: 0000-0002-6614-7158

*To whom correspondence may be addressed: Graduate School of Integrated Pharmaceutical and Nutritional Sciences, University of Shizuoka, Shizuoka, Japan, Tel.: +81-54-264-5576 (Ext.); Fax: +81-54-264-5576, E-mail: itosohei@u-shizuoka-ken.ac.jp

ORCID ID: 0000-0002-9937-3100

Contents

Supplementary Methods

Large-scape preparation and purification of chemoenzymatically synthesized EDMP for NMR analysis. S4

Determination of the yields of EDMP by chemical and chemoenzymatic methods. S4

Supplementary Figures

Schematic view of *tdh* and *kbl* operons in *Thermonaerovibrio acidaminovorans* DSM 6589, *Bacillus subtilis* subsp. subtilis str. 168, and *Vibrio cholerae* 01 biovar E1 Tor str. N16961. S5

GC-MS analysis of reaction product from L-Thr using CnTDH and CnKBL. S6

^{1}H NMR and ^{13}C NMR spectra for chemoenzymatically synthesized EDMP. S7

Calibration curve of EDMP in GC-MS. S8

Detection of Gly by LC-HRMS. S9

GC-MS analysis of reaction product from L-Thr using TaTDH and TaKBL. S10

Reaction scheme to evaluate TA activity of CnKBL. S11

Substrates used for UV-Vis spectra analysis of CnKBL. S12

Reaction scheme to evaluate KBL activity of CnKBL. S13

Characterization of KBL activity for CnKBL. S14

Superposed structures of CnKBL and EcKBL. S15

Superposed structures of CnKBL and eTA. S16

HPLC analysis of EDMP and DMP production with or without pre-reaction. S17

Comparison of the yield of EDMP and DMP with different timing of acetaldehyde addition. S18

MS spectrum for 2,3,5-trimethylpyrazine. S19

MS spectrum for 3-ethyl-2,5-dimethylpyrazine (EDMP). S20

MS spectrum for 2,5-dimethyl-3-propylpyrazine. S21

MS spectrum for 3-butyl-2,5-dimethylpyrazine. S22

MS spectrum for 3-isopentyl-2,5-dimethylpyrazine. S23

MS spectrum for 2,5-dimethyl-3-pentylpyrazine. S24

MS spectrum for 3-decyl-2,5-dimethylpyrazine. S25

MS spectrum for 3-benzyl-2,5-dimethylpyrazine. S26

GC-MS analysis of EDMP using aminoacetone and acetaldehyde at low concentration. S27

Supplementary Tables

Primers used in this study. S28
Annotation of TDH homologues. S29
Annotation of KBL homologues. S29
Enzymatic properties of CnKBL and EcKBL for KBL activity. S30
Enzymatic properties of CnKBL(WT) and two variants for TA activity. S30
Yields of EDMP and DMP under chemical or chemoenzymatic reactions. S31

Supplementary Methods

Large-scape preparation and purification of chemoenzymatically synthesized EDMP for NMR analysis.

The chemoenzymatic reaction was performed using 56.3 mM L-Thr, 37.5 mM NAD^+, and 100 mM potassium phosphate buffer (pH 8.0) at a total scale of 100 mL. To initiate the reaction, 0.05 mM CnKBL was added to reaction mixture. The reaction was performed for 6 h at 30 °C. After CnKBL reaction, 0.05 mM CnTDH was added to reaction mixture. The reaction was performed for 12 h at 30 °C. The produced EDMP was extracted three times with diethyl ether. The combined organic layers were dried Na_2SO_4 and the solvents were removed under reduced pressure. Silica-gel column chromatography was carried out using silica gel 60N (230–400 mesh, Kanto Chemical, Tokyo, Japan). The isolated EDMP was analyzed by nuclear magnetic resonance (NMR). NMR spectra were acquired on a Bruker Biospin AVANCE-III (400 MHz) spectrometer (Bruker BioSpin, Rheinstetten, Germany), with chemical shifts expressed in ppm. **TLC** (hexane/diethyl ether 1:1): R_f = 0.31. **^{1}H NMR** (400 MHz, $CDCl_3$): δ = 1.26 (t, J = 7.6 Hz, 3H), 2.48 (s, 3H), 2.52 (s, 3H), 2.79 (q, J = 7.6 Hz, 2H), 8.14 (s, 1H). **^{13}C NMR** (100 MHz, $CDCl_3$): δ = 12.6, 21.0 (2C), 28.2, 140.6, 148.3, 150.1, 155.7. ^{1}H NMR and ^{13}C NMR spectra for EDMP are shown in Supplementary Fig. 3.

Determination of the yields of EDMP by chemical and chemoenzymatic methods.

The reaction conditions are basically the same as in the sections "Enzymatic reaction using CnTDH and CnKBL" and "Chemoenzymatic reaction using CnTDH and aldehydes " in the main text. In brief, the chemical reactions were carried out using 5.0 mM aminoacetone and 2.5 mM acetaldehyde. In the chemoenzymatic reaction, the enzymes were reacted with 2.5-7.5 mM L-Thr with or without chemical reagents. The term "pre-reaction" is defined as the condensation of aminoacetone in the absence of acetaldehyde to form an intermediate. These methods were used in Supplementary Figure 13, Figure 14, and Supplementary Table 6.

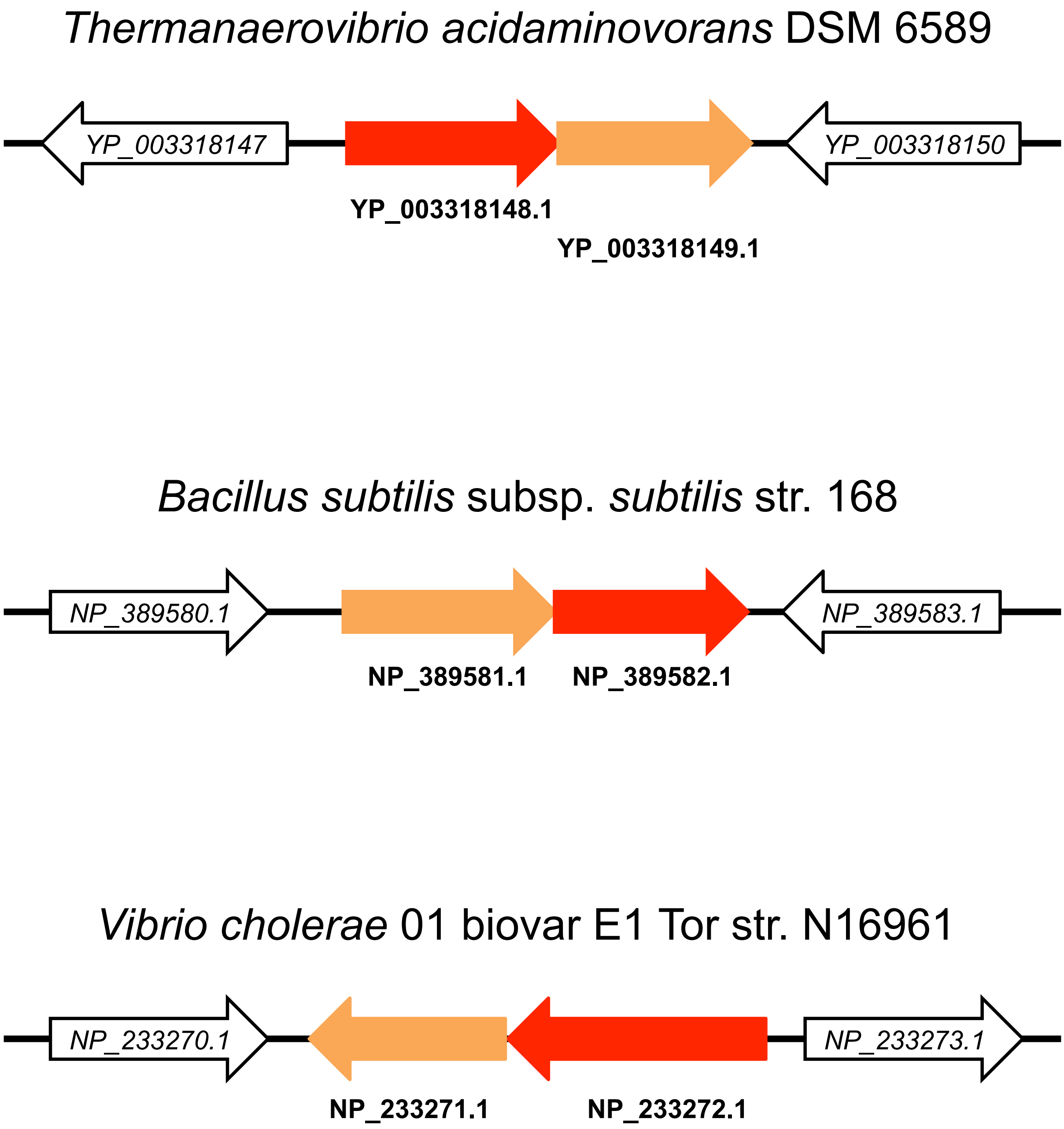

Supplementary Fig. 1 Schematic view of *tdh* and *kbl* operons in *Thermonaerovibrio acidaminovorans* DSM 6589, *Bacillus subtilis* subsp. *subtilis* str. 168, and *Vibrio cholerae* 01 biovar E1 Tor str. N16961.

tdh and *kbl* genes are colored by orange and red, respectively.

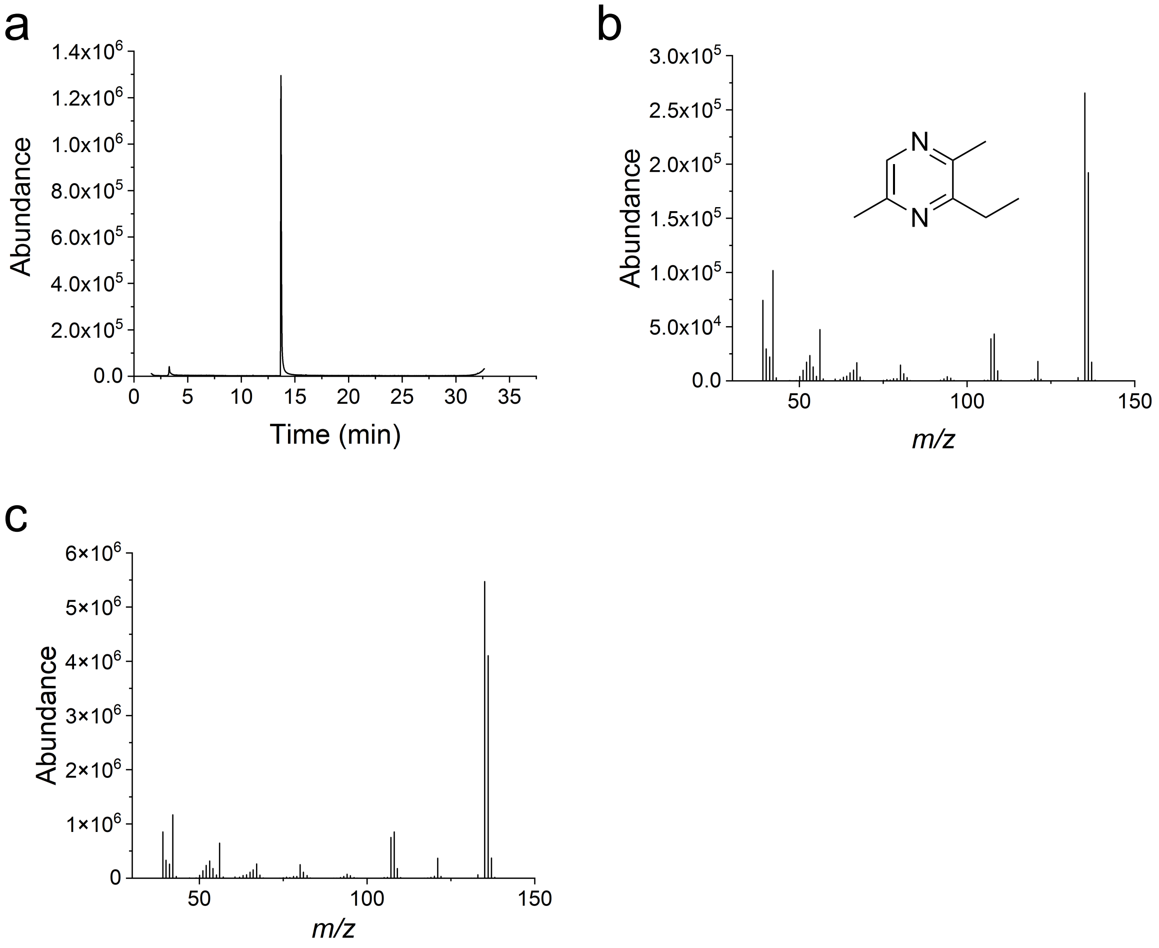

Supplementary Fig. 2 GC-MS analysis of reaction product from L-Thr using CnTDH and CnKBL.

Chromatogram and mass spectrum are shown (a) and (b), respectively. Enzyme reaction using purified CnTDH and CnKBL was performed using assay buffer A [100 mM potassium phosphate (pH 8.0), 10 mM L-Thr and 5 mM NAD^+]. To start the reaction, 0.05 mM CnTDH and CnKBL were added to assay buffer A. The enzyme reaction was performed for 3 h at 30°C. After the enzyme reaction, reaction mixture was provided to a GC-MS analysis. The *m/z* of EDMP standard is shown (c). Comparison of reaction product to the EDMP standard indicated that reaction product is EDMP.

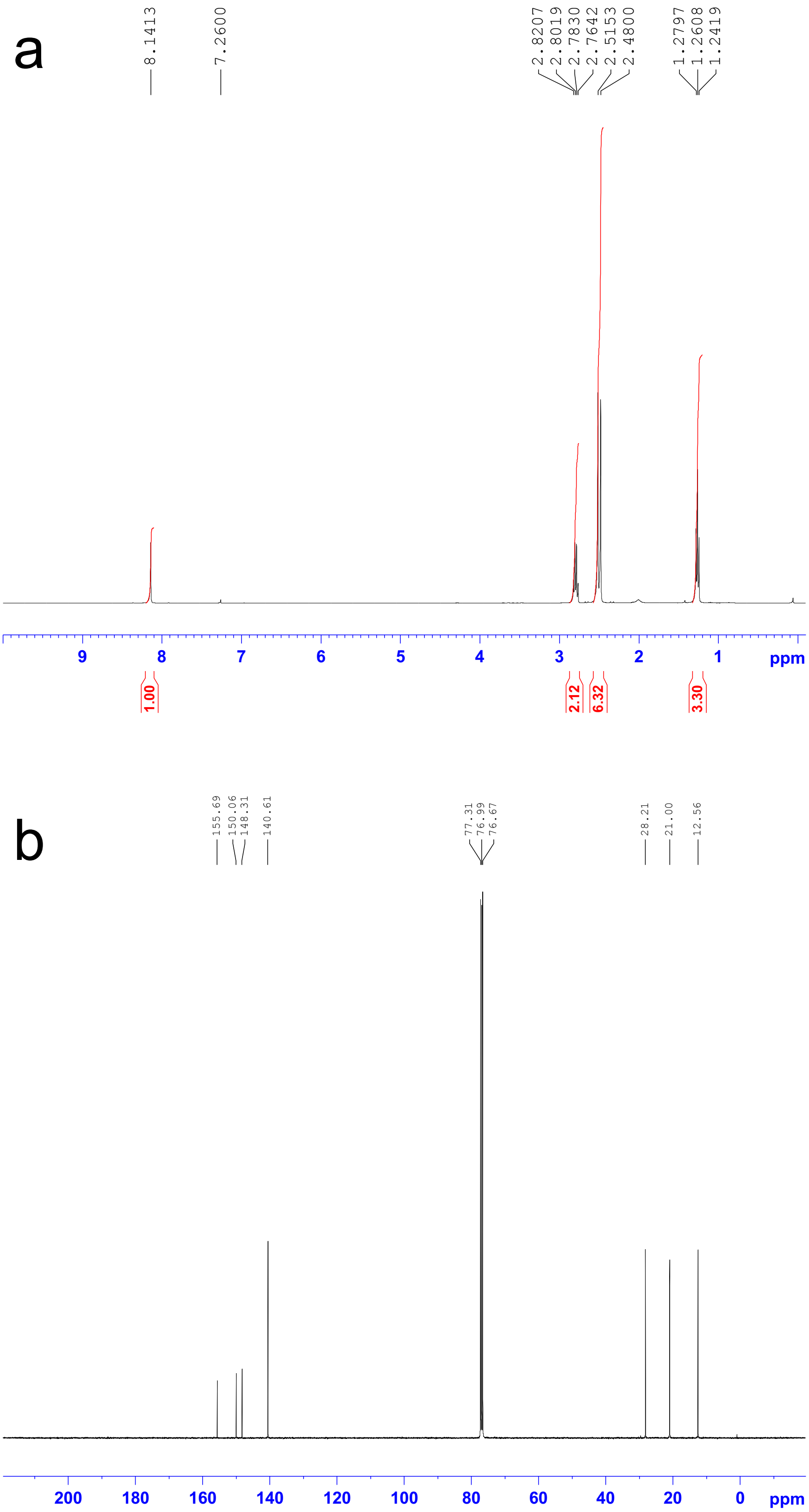

Supplementary Fig. 3 ^{1}H NMR and ^{13}C NMR spectra for chemoenzymatically synthesized EDMP.

^{1}H NMR and ^{13}C NMR spectra for EDMP are shown (a) and (b), respectively.

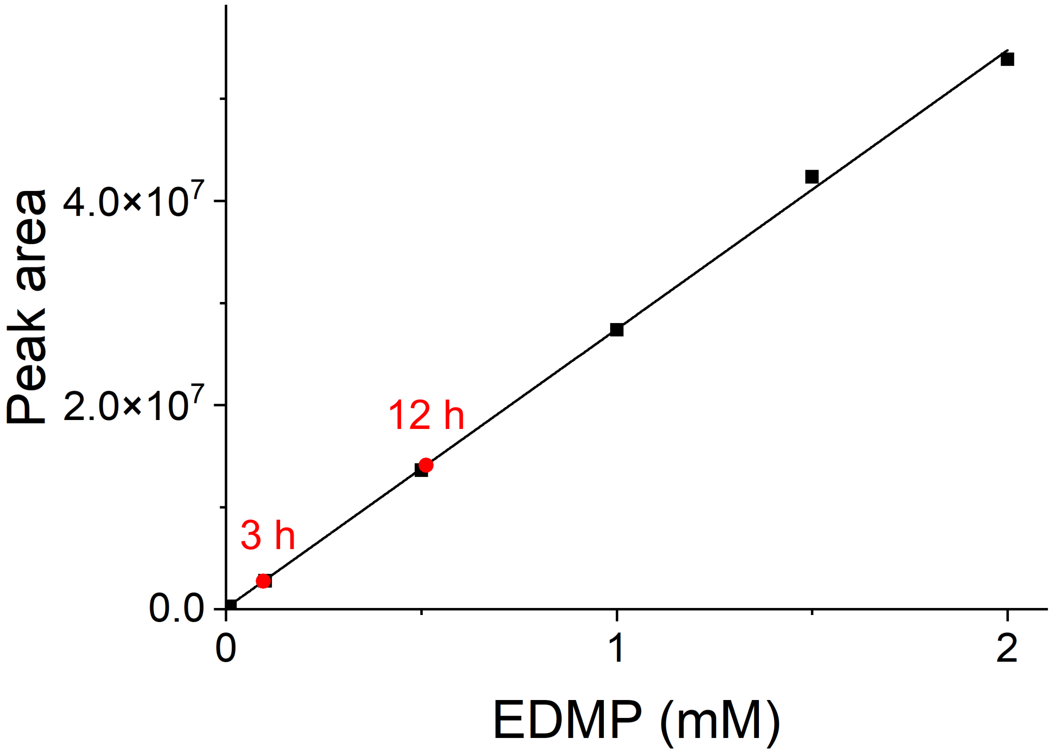

Supplementary Fig. 4 Calibration curve of EDMP in GC-MS.

Calibration curve was obtained using 0.01–2.0 mM EDMP standard. EDMP standard and produced EDMP using CnTDH and CnKBL from L-Thr (reaction time: 3 and 12 h) are shown black and red, respectively.

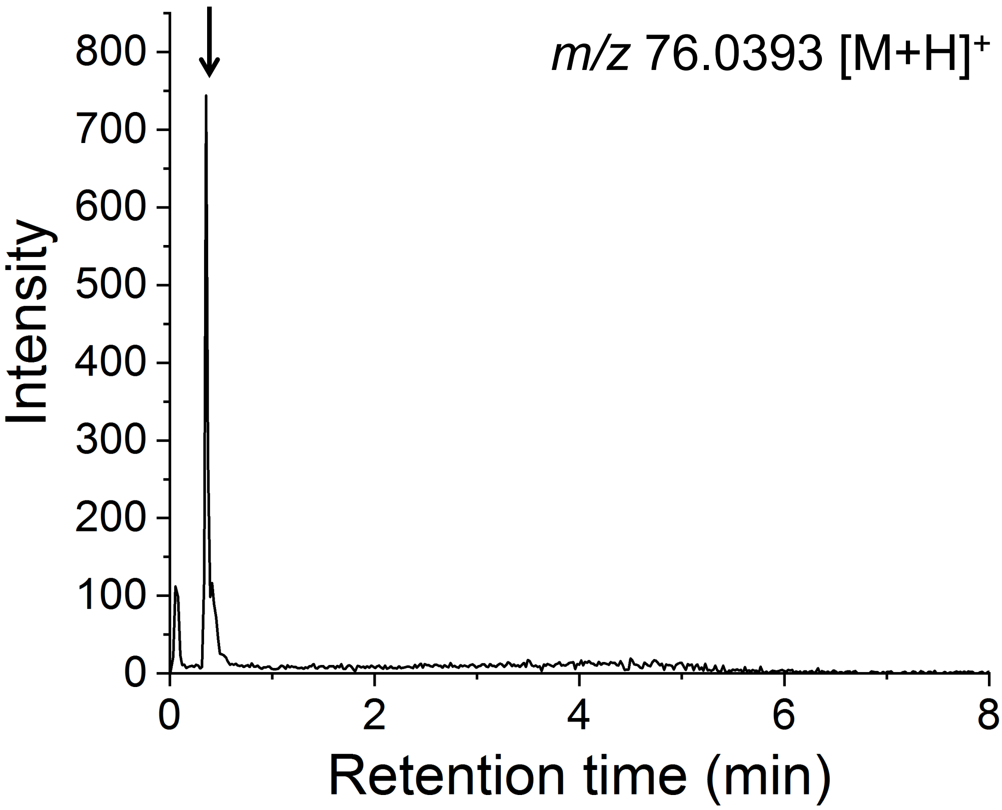

Supplementary Fig. 5 Detection of Gly by LC-HRMS.

Extracted ion count chromatograms of *m/z* 76.0393 ± 0.0020 $[M+H]^+$ are shown. The peak of Gly is represented as an arrow.

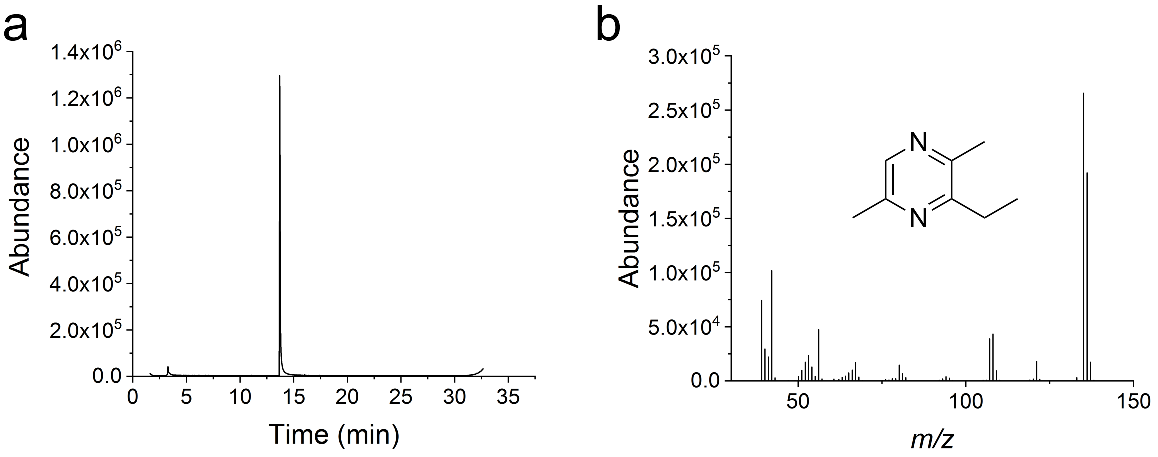

Supplementary Fig. 6 GC-MS analysis of reaction product from L-Thr using TaTDH and TaKBL.

Chromatogram and mass spectrum are shown (a) and (b), respectively. Enzyme reaction using purified TaTDH and TaKBL was performed using assay buffer A [100 mM potassium phosphate (pH 8.0), 10 mM L-Thr and 5 mM NAD^+]. To start the reaction, 0.05 mM TaTDH and TaKBL were added to assay buffer A. The enzyme reaction was performed for 3 h at 30°C. After the enzyme reaction, reaction mixture was provided to a GC-MS analysis.

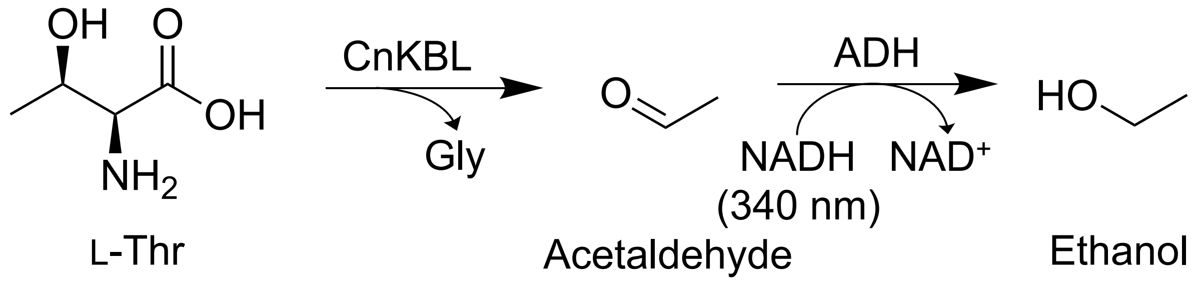

Supplementary Fig. 7 Reaction scheme to evaluate TA activity of CnKBL.

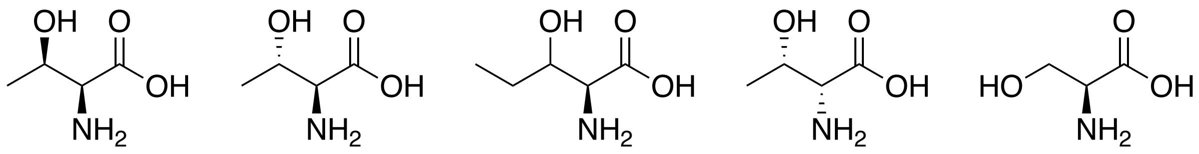

L-Thr L-all-Thr DL-3-HN D-Thr L-Ser

Supplementary Fig. 8 Substrates used for UV-Vis spectra analysis of CnKBL.

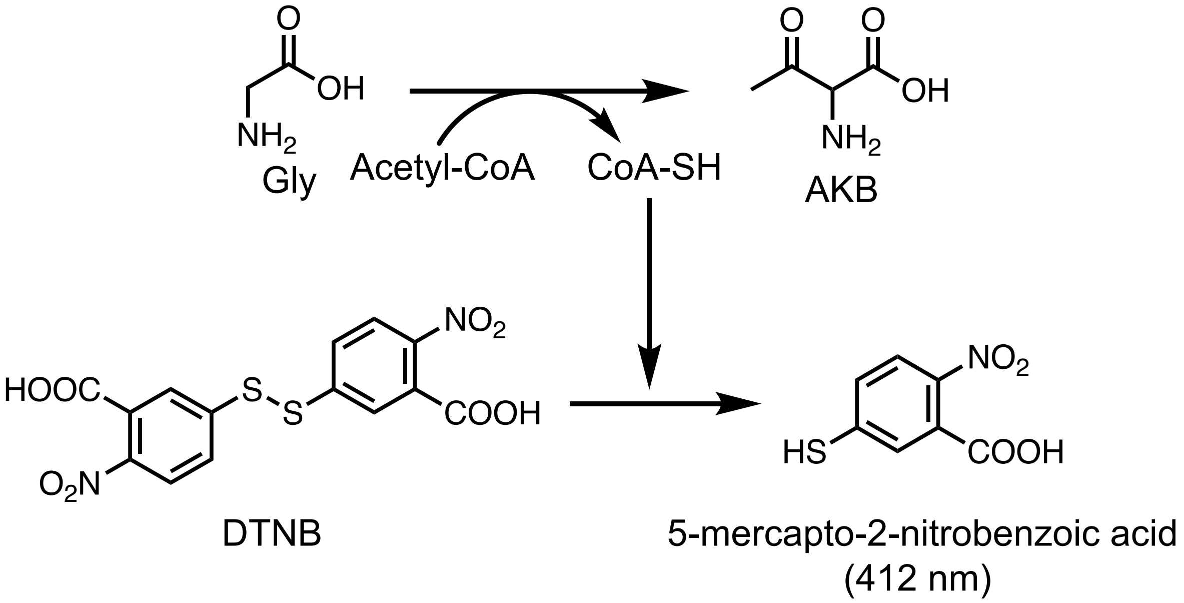

Supplementary Fig. 9 Reaction scheme to evaluate KBL activity of CnKBL.

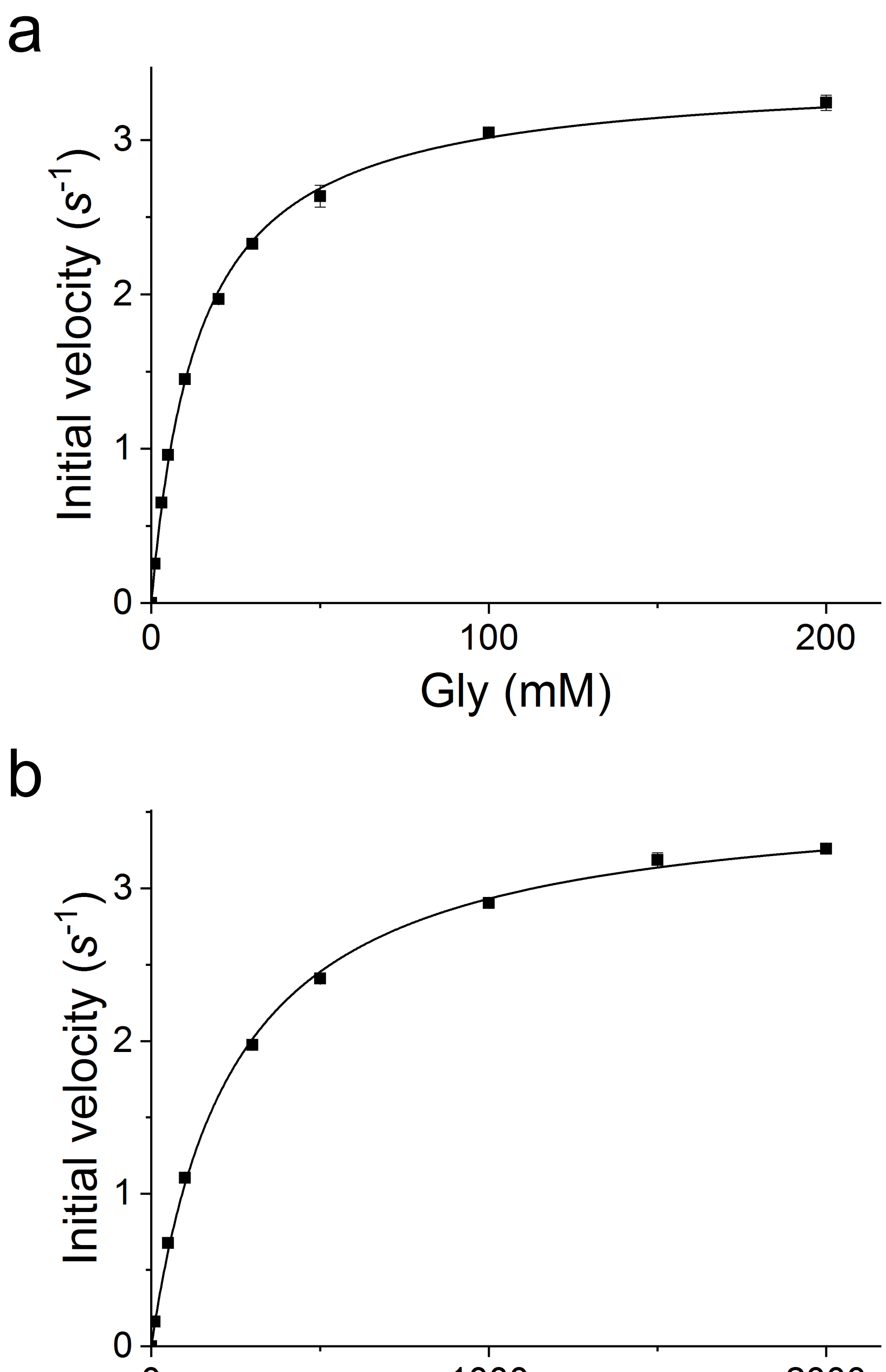

Supplementary Fig. 10 Characterization of KBL activity for CnKBL.

Enzyme kinetic plots of KBL activity of CnKBL toward Gly (a) and Acetyl-CoA (b). The data are represented as mean ± standard deviation. Kinetic parameters are listed in Supplementary Table 4.

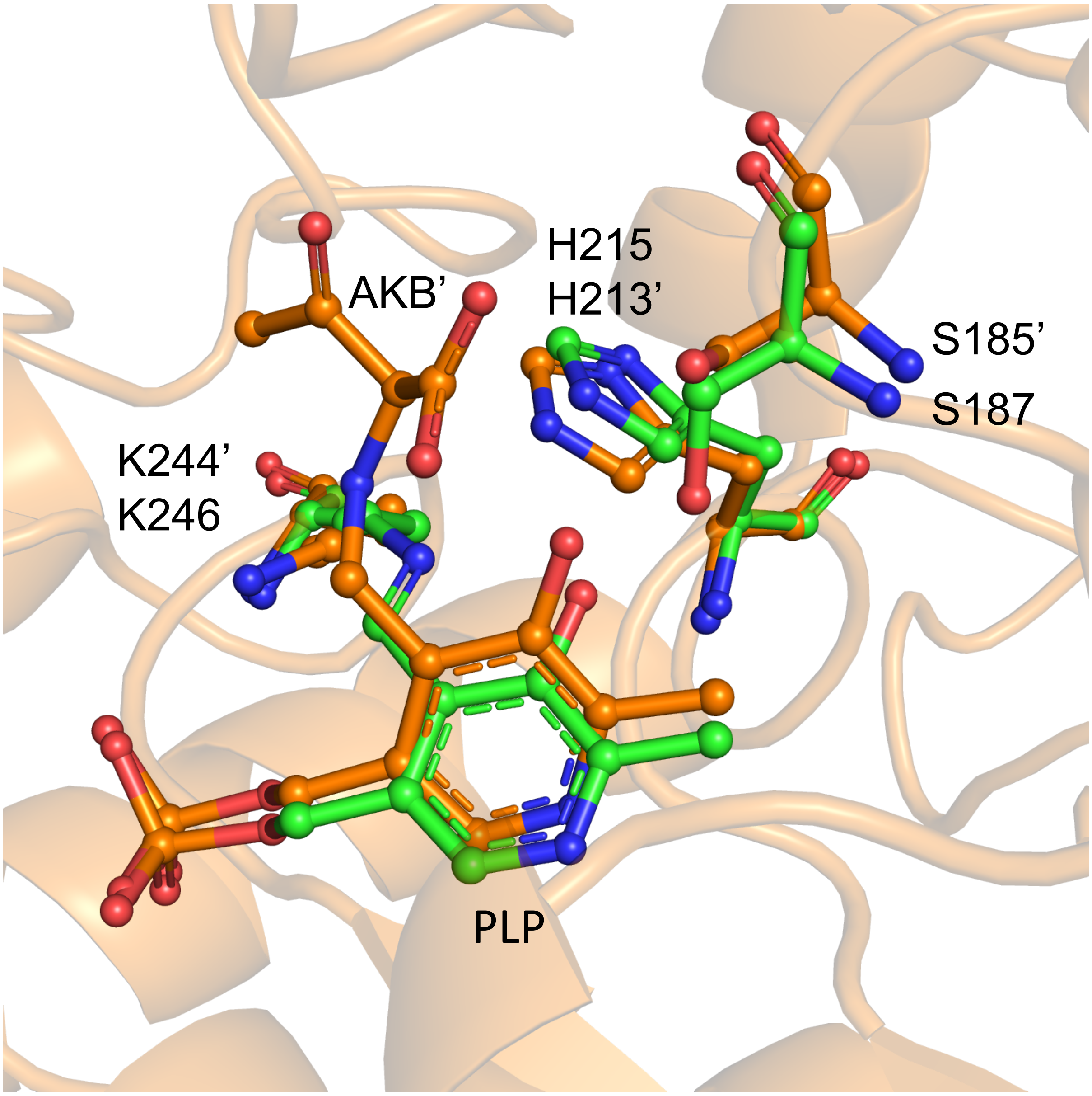

Supplementary Fig. 11 Superposed structures of CnKBL and EcKBL.

CnKBL and EcKBL (PDB entry 1FC4) are colored green and orange, respectively. Residues in EcKBL are marked by using single-quotation. Conserved residues between CnKBL and EcKBL are represented as stick model.

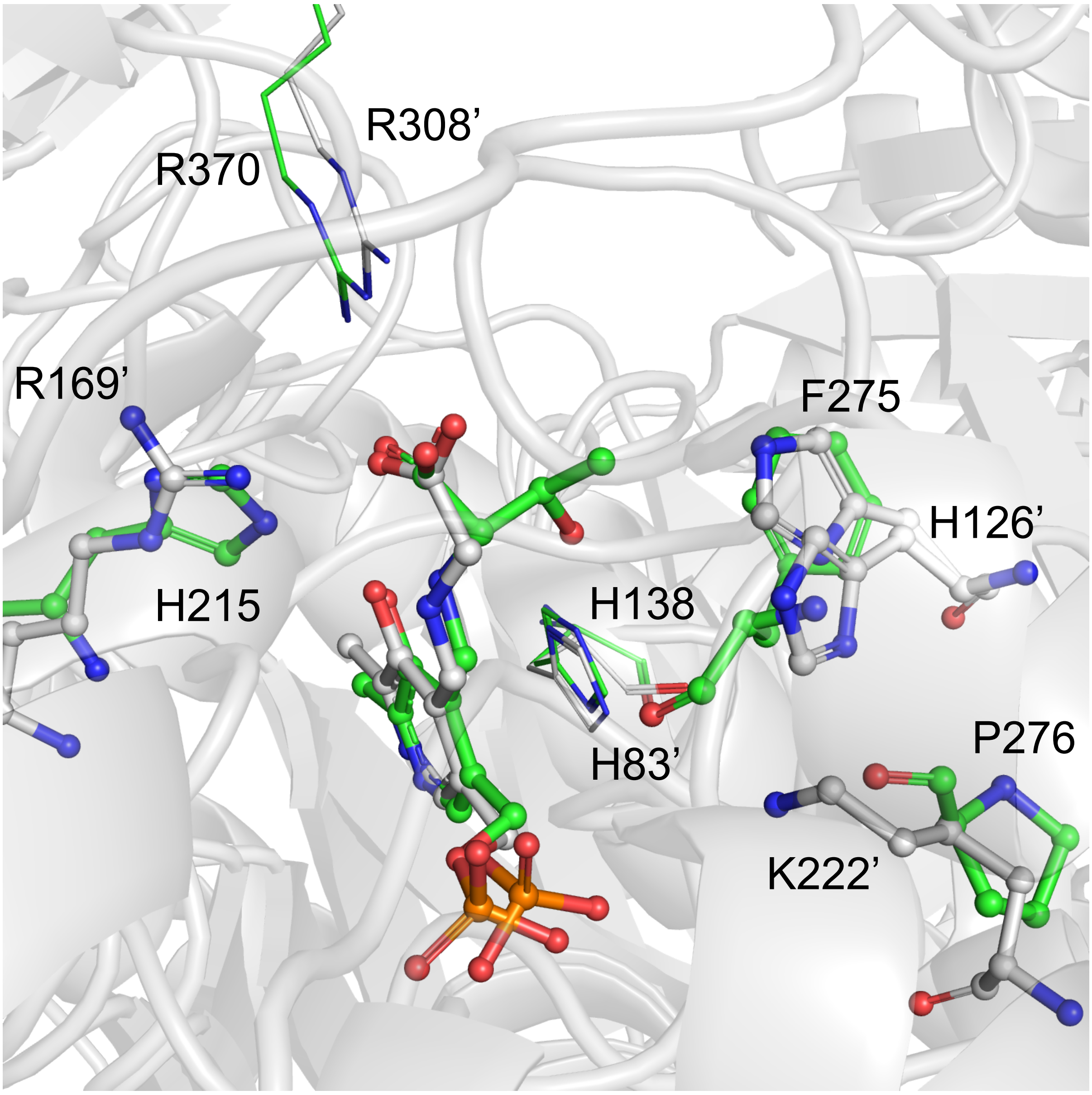

Supplementary Fig. 12 Superposed structures of CnKBL and eTA.

CnKBL and eTA (PDB entry 4LNM) are colored green and gray, respectively. Residues in eTA are marked by using single-quotation. Conserved residues between CnKBL and eTA are represented as line model. Different residues between CnKBL and eTA are represented as stick model. H138, H215, F275, Lys222 and R370 in CnKBL are corresponding to H83, R169, H126, P276 and R308 in eTA, respectively.

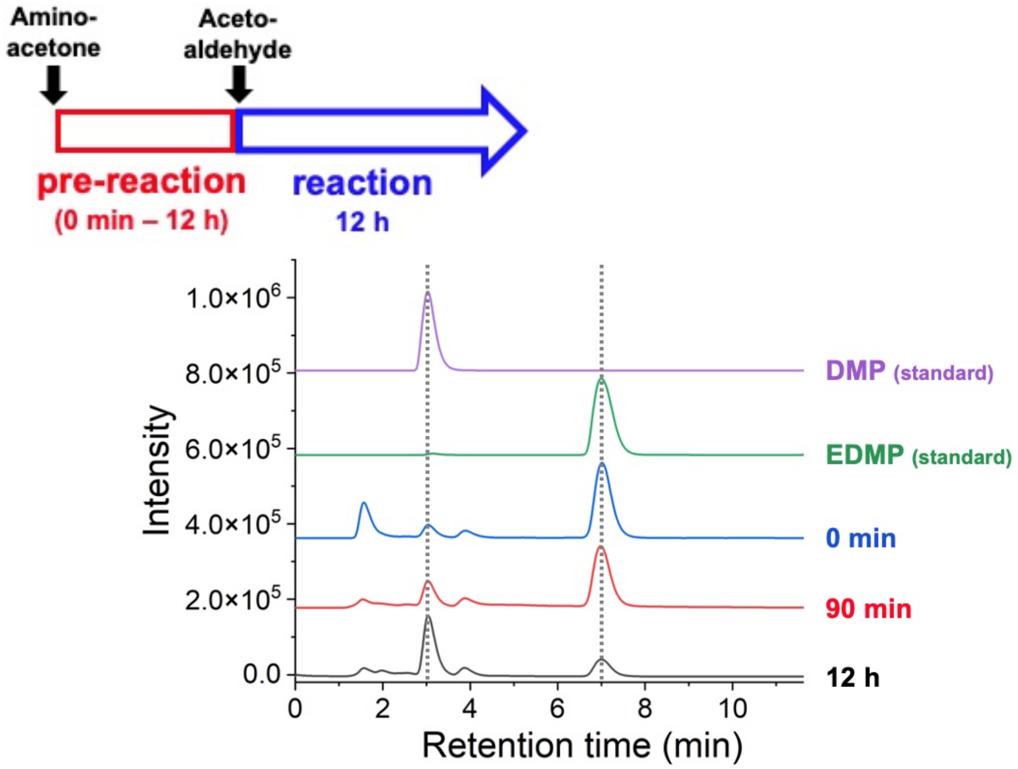

Supplementary Fig. 13 HPLC analysis of EDMP and DMP production with or without pre-reaction.

The EDMP and DMP standard are colored green and purple line, respectively. HPLC analysis was performed after the reaction of aminoacetone and acetaldehyde.

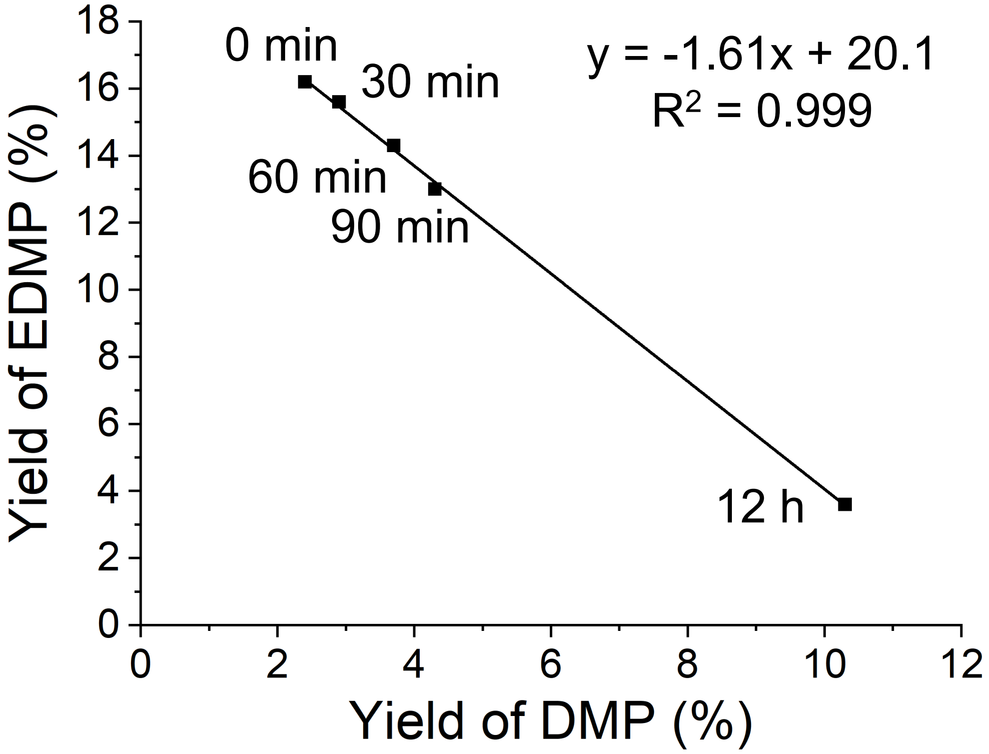

Supplementary Fig. 14 Comparison of the yield of EDMP and DMP with different timing of acetaldehyde addition.

This figure was generated from the values shown in entry 5–9 in Supplemental Table 6.

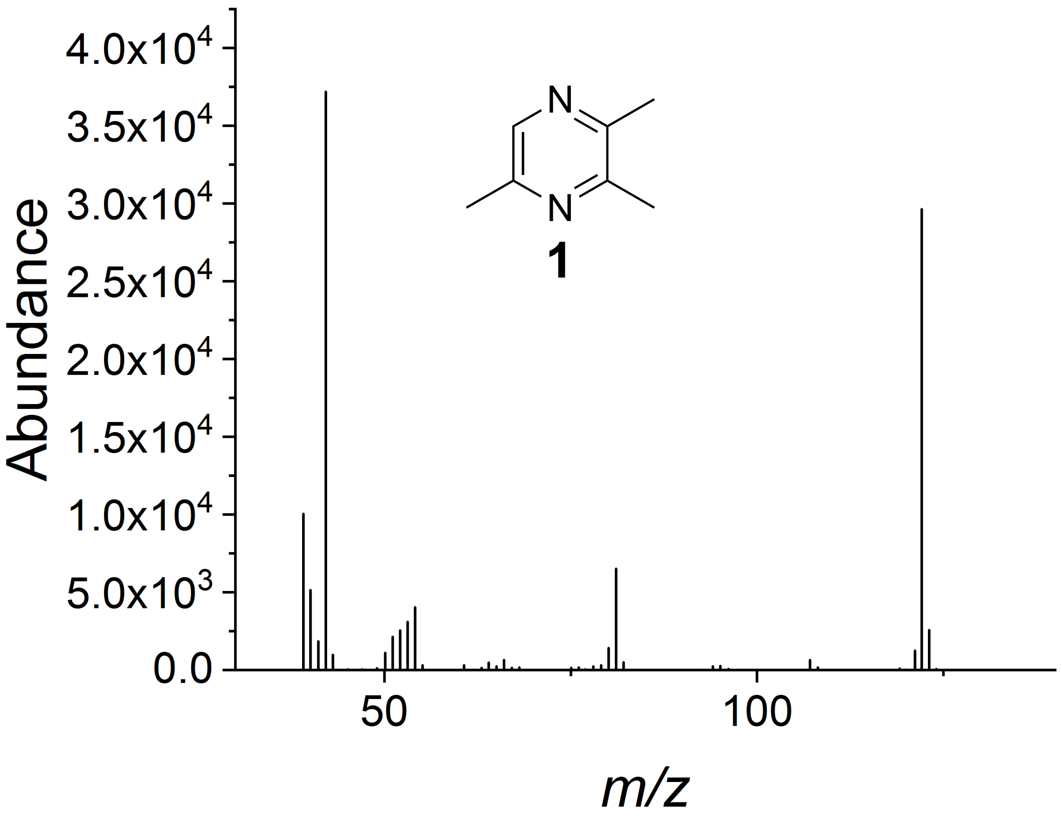

Supplementary Fig. 15 MS spectrum for 2,3,5-trimethylpyrazine.

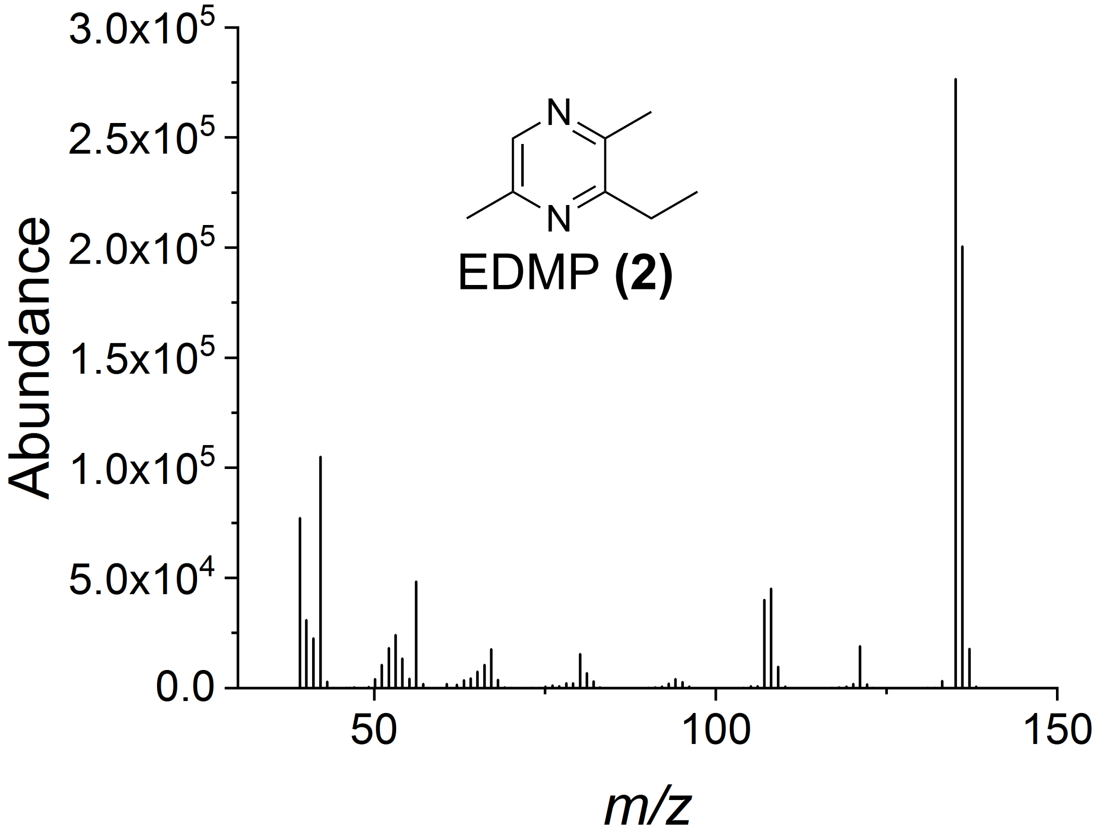

Supplementary Fig. 16 MS spectrum for 3-ethyl-2,5-dimethylpyrazine (EDMP).

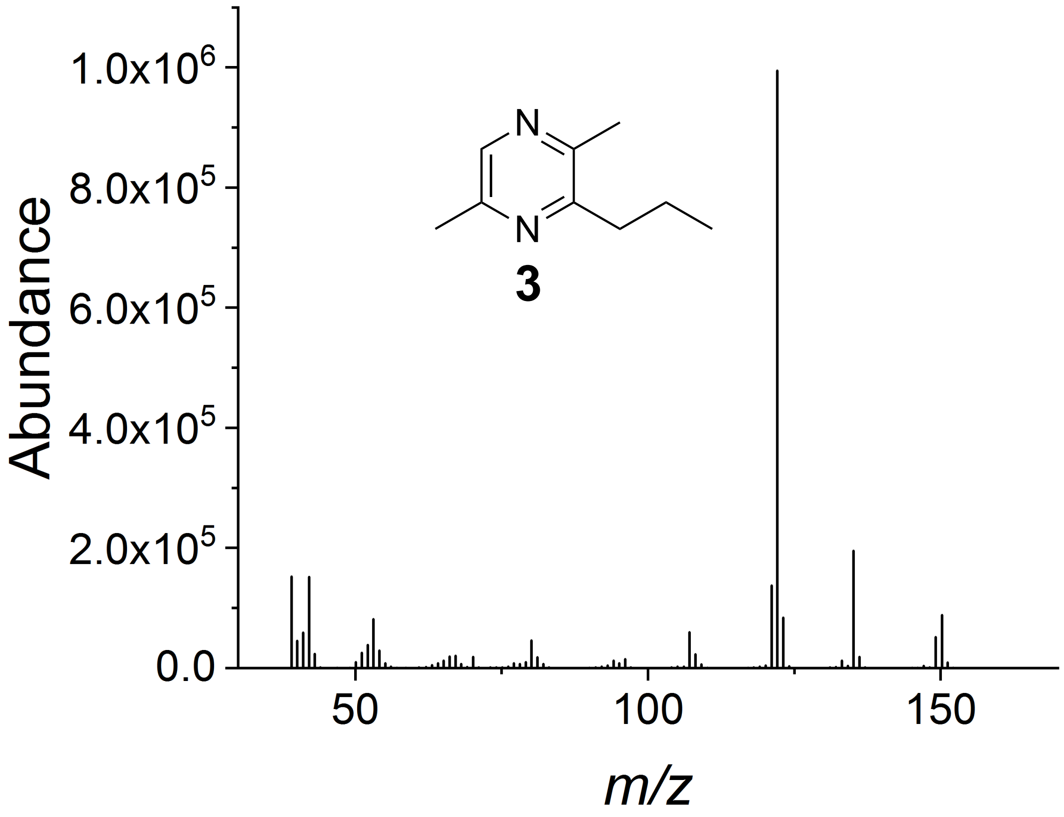

Supplementary Fig. 17 MS spectrum for 2,5-dimethyl-3-propylpyrazine.

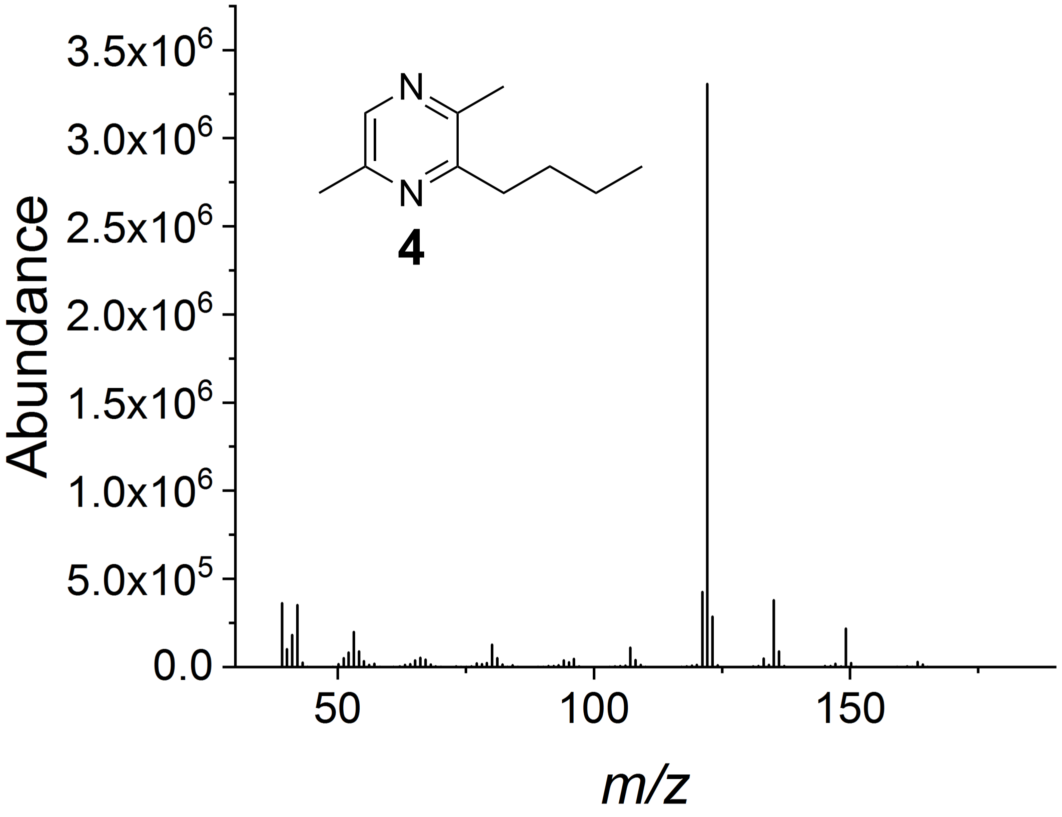

181
182 **Supplementary Fig. 18 MS spectrum for 3-butyl-2,5-dimethylpyrazine.**

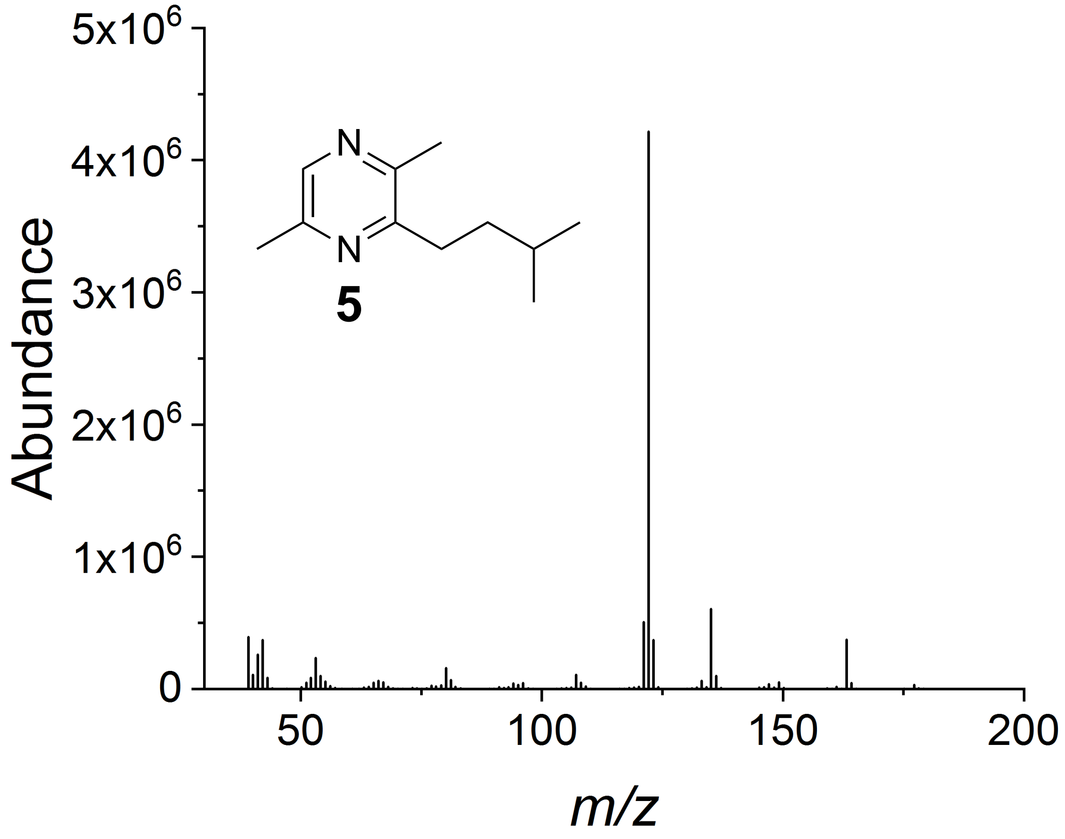

Supplementary Fig. 19 MS spectrum for 3-isopentyl-2,5-dimethylpyrazine.

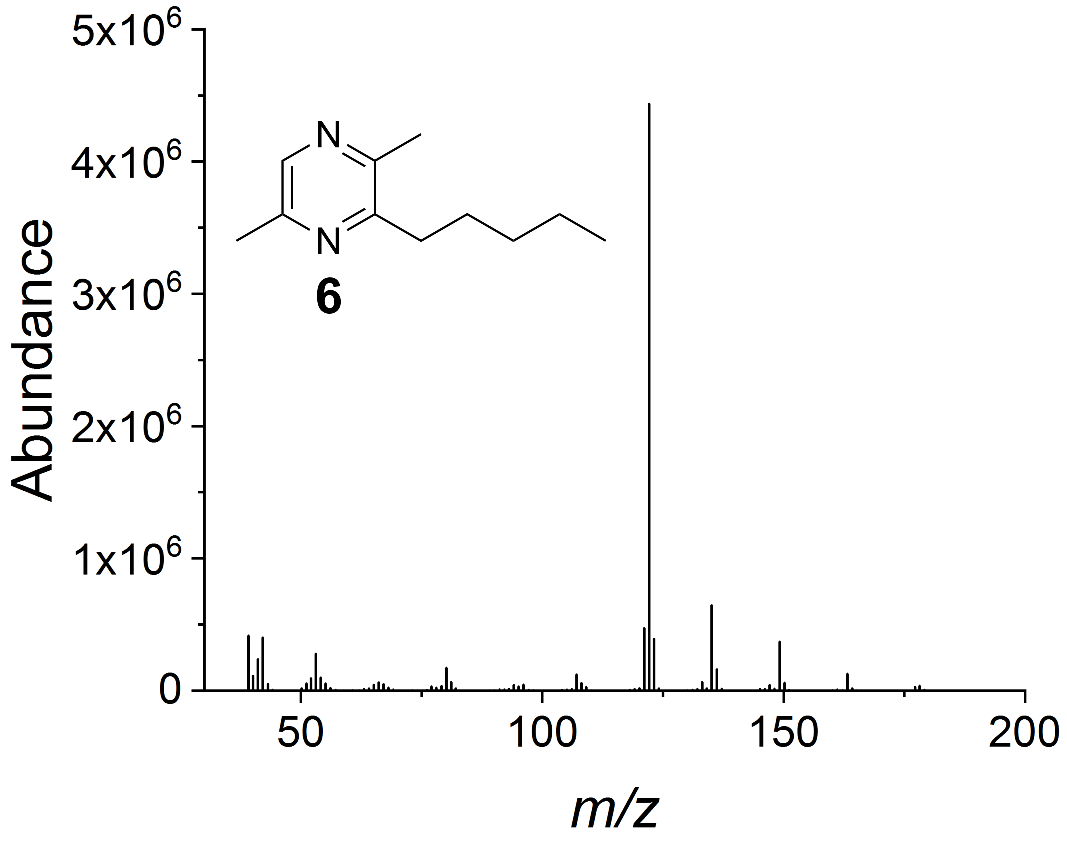

Supplementary Fig. 20 MS spectrum for 2,5-dimethyl-3-pentylpyrazine.

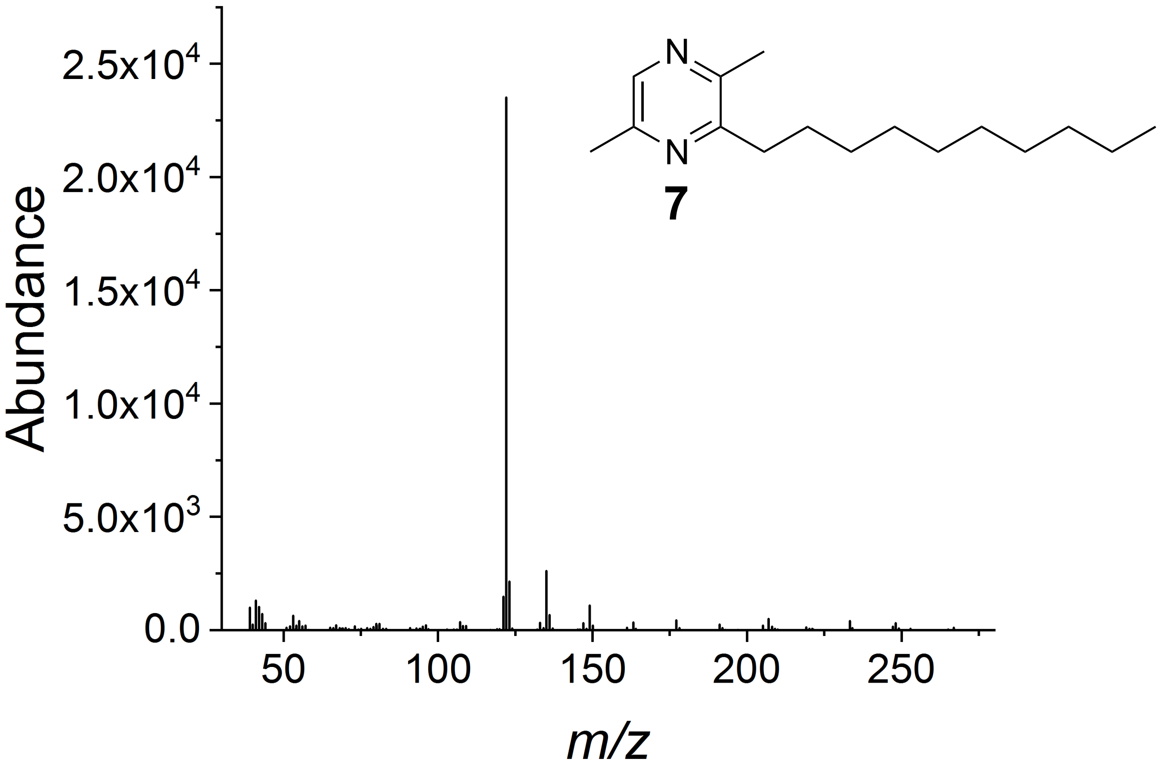

Supplementary Fig. 21 MS spectrum for 3-decyl-2,5-dimethylpyrazine.

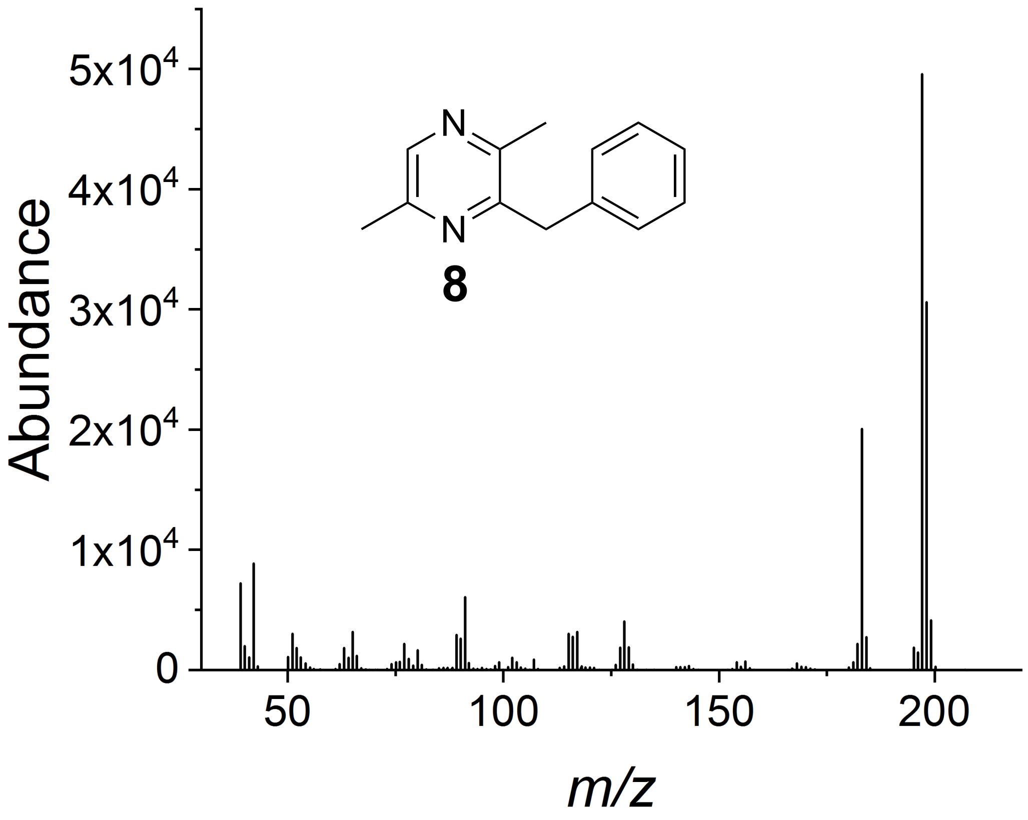

Supplementary Fig. 22 MS spectrum for 3-benzyl-2,5-dimethylpyrazine.

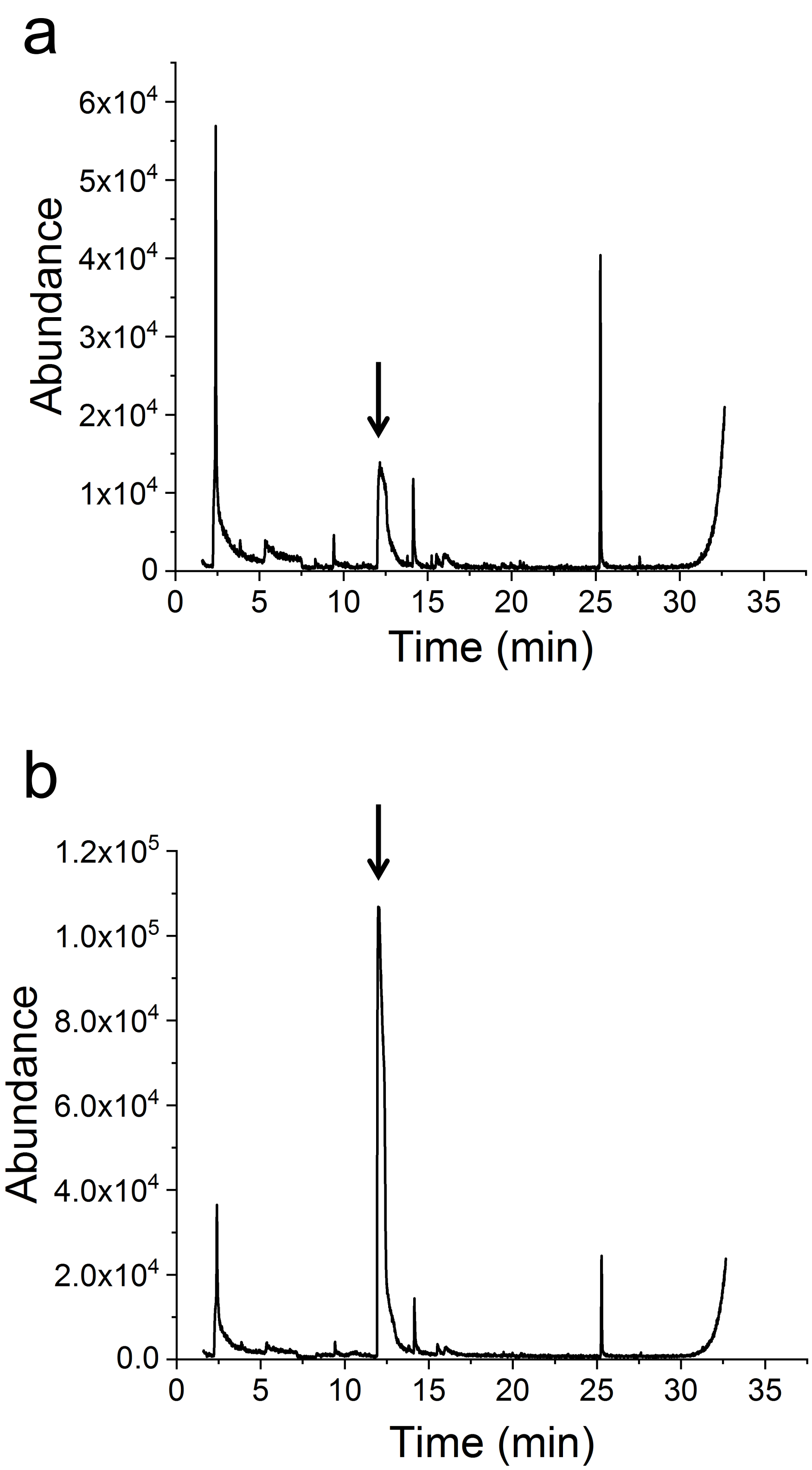

Supplementary Fig. 23 GC-MS analysis of EDMP using aminoacetone and acetaldehyde at low concentration.

The peaks can be detected in the reaction condition containing 500 nM (a) and 5 μM (b) aminoacetone and acetaldehyde. The peak of EDMP is represented as an arrow.

Supplementary Table 1 Primers used in this study.

Primer	Sequence (5'→3')
*cnkbl*_F	ATATCCATGGGCCATATGATGTCGAATGCCGAGGC
*cnkbl*_R	ATATGGATCCTTACTCGAGGATCAGCCCCAGTTCAC
H138F	ATCAGCGATGCGCTCAAC**TTT**GCCTCGATCATCGAC
K246A	ATCATTACCGGCACGCTGGGC**GCG**GCGCTGGGTGGC

Supplementary Table 2 Annotation of TDH homologues.

Accession	Definition	Source
WP_010813492.1	L-threonine 3-dehydrogenase	*Cupriavidus necator*
YP_003318149.1	NAD-dependent epimerase/dehydratese	*Thermanaerovibrio acidaminovorans* DSM 6589
NP_389581.1	threonine 3-dehydrogenase	*Bacillus subtilis* subsp. *subtilis* str. 168
NP_233271.1	L-threonine 3-dehydrogenase	*Vibrio cholerae* 01 biovar E1 Tor str. N16961

Supplementary Table 3 Annotation of KBL homologues.

Accession	Definition	Source
WP_011616818.1	glycine C-acetyltransferase	*Cupriavidus necator*
YP_003318148.1	pyridoxal phosphate-dependent acyltransferase	*Thermanaerovibrio acidaminovorans* DSM 6589
NP_389582.1	2-amino-3-ketobutyrate CoA ligase (glycine acetyl transferase)	*Bacillus subtilis* subsp. *subtilis* str. 168
NP_233272.1	2-amino-3-ketobutyrate CoA ligase	*Vibrio cholerae* 01 biovar E1 Tor str. N16961

Supplementary Table 4 Enzymatic properties of CnKBL and EcKBL for KBL activity.

	k_{cat} (s^{-1})[a]	$K_{m, Gly}$ (mM)[b]	$K_{m, Acetyl\text{-}CoA}$ (μM)[b]	$k_{cat}/K_{m, Gly}$ (sec^{-1} mM^{-1})
CnKBL	3.43 ± 0.036	13.9 ± 0.52	242.7 ± 10.4	0.25
EcKBL	1.9[c]	12.0[c]	59[c]	0.16[c]

[a] k_{cat} value of CnKBL for KBL activity were derived from the data shown in Supplementary Fig. 10.

[b] $K_{m, Gly}$ and $K_{m, Acetyl\text{-}CoA}$ values represent the Michaelis constant value toward Gly and Acetyl-CoA, respectively.

[c] Mukherjee, J. J.; Dekker, E. E., Purification, properties, and N-terminal amino acid sequence of homogeneous Escherichia coli 2-amino-3-ketobutyrate CoA ligase, a pyridoxal phosphate-dependent enzyme. *J Biol Chem* **1987,** *262* (30), 14441-7.

Supplementary Table 5 Enzymatic properties of CnKBL(WT) and two variants for TA activity.

	k_{cat} (min^{-1})[a]	K_m (mM)[b]	k_{cat}/K_m (min^{-1} mM^{-1})
WT	0.170 ± 0.39	1.24 ± 0.11	0.14
H138F	n.d.[c]	n.d.	n.d.
K246A	n.d.	n.d.	n.d.

[a] k_{cat} values of CnKBL for TA activity were derived from the data shown in Fig. 4b.

[b] K_m values represent the Michaelis constant value toward L-Thr.

[c] n.d., not determined.

Supplementary Table 6 Yields of EDMP and DMP under chemical or chemoenzymatic reactions.

Entry	Reaction condition	Yield of EDMP (%) (Yield of DMP)[a]				
		Reaction time				
		1 h	2 h	3 h	6 h	12 h
Chemical reaction (Using aminoacetone and acetaldehyde reagents)						
1	30°C	-	-	-	-	16.2
2	40°C	-	-	-	-	13.1
3	50°C	-	-	-	-	9.9
4	60°C	-	-	-	-	3.7
Chemical reaction with pre-reaction (30°C)						
5	Without pre-reaction	7.6 (1.4)	11.0 (1.4)	12.3 (1.9)	14.8 (2.4)	16.2 (2.4)
6	30 min pre-reaction	7.7 (2.4)	10.5 (3.0)	11.9 (3.5)	14.4 (4.8)	15.6 (2.9)
7	60 min pre-reaction	8.3 (3.4)	10.9 (3.6)	11.3 (3.4)	12.8 (4.8)	14.3 (3.7)
8	90 min pre-reaction	6.3 (3.8)	9.4 (4.1)	10.3 (4.7)	11.4 (5.7)	13.0 (4.3)
9	12 h pre-reaction	1.4 (9.9)	2.0 (10.2)	2.3 (10.1)	3.2 (10.5)	3.6 (10.3)
Chemoenzymatic reaction (30°C)						
10	TDH and acetaldehyde (Aminoacetone was provided from L-Thr)	1.1 (0.3)	4.3 (0.4)	7.6 (0.6)	15.2 (1.3)	23.4 (2.1)
11	KBL and aminoacetone (Acetaldehyde was provided from L-Thr)	1.0 (2.5)	3.1 (3.3)	5.1 (3.9)	8.2 (4.6)	10.8 (5.2)
12	TDH and KBL (Using L-Thr as substrate)	0.2 (0.5)	1.6 (1.5)	3.9 (2.5)	10.7 (4.0)	20.2 (5.6)

[a] The yield of DMP was shown in the parentheses.
