# Supplementary material for: Chemoenzymatic synthesis of 3-ethyl-2,5-dimethylpyrazine by L-threonine 3-dehydrogenase and 2-amino-3-ketobutyrate CoA ligase/L-threonine aldolase
Source: Commun Chem. 2021 Jul 16;4:108. doi: 10.1038/s42004-021-00545-8 (PMC9814548; doi:10.1038/s42004-021-00545-8)
Supplement: Supplementary file 2 — Supplementary Data 1 [file 42004_2021_545_MOESM2_ESM.docx]

>CnTDH

MEAGKPKILIVGANGQIGSELALALAERYGRTNVITSDVVPTGRHVHLTHEMLNATDRGELATVVERHGITQVYLLAAALSATGEKAPQWAWNLNMTSLLNVLELARQTGLERVFWPSSIAAFGPTTPAGQTPQKTVMEPTTVYGISKQAGEGWCRWYHANHGVDVRSVRYPGLISHKTPPGGGTTDYAVDIFHAAVTGEPYTCFLKEDEALPMMYMPDAIRATIELMEAPADKLSERGSYNIAGMSFTPAQIAAAIREQVPGFQIRYEPDYRQAIAQGWPDSIDDSVARADWGWKAQYGLKEMVADMLANLKATLAG

>CnKBL

MSNAEAFYASIRTELESIRAAGLFKNERVIATPQGARVRTTDGREVINLCANNYLGLSSHPQVIEAAHEALRTHGFGLSSVRFICGTQDLHKTLEARLSAFLGTEDTILYGSAFDANGGLFETLLGAEDAVISDALNHASIIDGVRLSKARRYRYQHNDMDDLRVQLEQARADGARYTLVFSDGVFSMDGTVARLDEMRAICDEYGALLGIDECHATGFMGQRGRGTHEARGVFGKIDIITGTLGKALGGASGGFTSARKEVVALLRQRSRPYLFSNTVAPAIVGASIAVLDILEASTELRDRLEGNTRFFRAGLDRLGFDVKAGDHPIIPIMVYDADKAQQLAQRLLELGVYVVGFFYPVVPKGQARIRVQMSALHDEAALQAALDAFGQAGRELGLI
