# Supplementary material for: Chemoenzymatic synthesis of 3-ethyl-2,5-dimethylpyrazine by L-threonine 3-dehydrogenase and 2-amino-3-ketobutyrate CoA ligase/L-threonine aldolase
Source: Commun Chem. 2021 Jul 16;4:108. doi: 10.1038/s42004-021-00545-8 (PMC9814548; doi:10.1038/s42004-021-00545-8)
Supplement: Supplementary file 4 — Supplementary Data 3 [file 42004_2021_545_MOESM4_ESM.pdf]

Full wwPDB X-ray Structure Validation Report (i)

Apr 20, 2021 – 08:31 AM JST

PDB ID	:	7BXP
Title	:	2-amino-3-ketobutyrate CoA ligase from Cupriavidus necator
Authors	:	Motoyama, T.; Nakano, S.; Hasebe, F.; Miyoshi, N.; Ito, S.
Deposited on	:	2020-04-20
Resolution	:	1.80 Å(reported)

This is a Full wwPDB X-ray Structure Validation Report for a publicly released PDB entry.

We welcome your comments at *validation@mail.wwpdb.org*
A user guide is available at
https://www.wwpdb.org/validation/2017/XrayValidationReportHelp
with specific help available everywhere you see the (i) symbol.

The following versions of software and data (see references (i)) were used in the production of this report:

MolProbity	:	4.02b-467
Mogul	:	1.8.5 (274361), CSD as541be (2020)
Xtriage (Phenix)	:	1.13
EDS	:	2.18
Percentile statistics	:	20191225.v01 (using entries in the PDB archive December 25th 2019)
Refmac	:	5.8.0158
CCP4	:	7.0.044 (Gargrove)
Ideal geometry (proteins)	:	Engh & Huber (2001)
Ideal geometry (DNA, RNA)	:	Parkinson et al. (1996)
Validation Pipeline (wwPDB-VP)	:	2.18

1 Overall quality at a glance (i)

The following experimental techniques were used to determine the structure:
X-RAY DIFFRACTION

The reported resolution of this entry is 1.80 Å.

Percentile scores (ranging between 0-100) for global validation metrics of the entry are shown in the following graphic. The table shows the number of entries on which the scores are based.

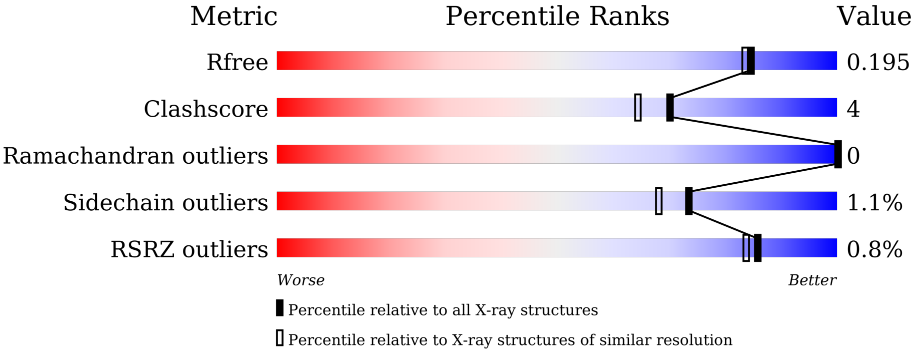

Metric	Whole archive (#Entries)	Similar resolution (#Entries, resolution range(Å))
R_{free}	130704	5950 (1.80-1.80)
Clashscore	141614	6793 (1.80-1.80)
Ramachandran outliers	138981	6697 (1.80-1.80)
Sidechain outliers	138945	6696 (1.80-1.80)
RSRZ outliers	127900	5850 (1.80-1.80)

The table below summarises the geometric issues observed across the polymeric chains and their fit to the electron density. The red, orange, yellow and green segments of the lower bar indicate the fraction of residues that contain outliers for >=3, 2, 1 and 0 types of geometric quality criteria respectively. A grey segment represents the fraction of residues that are not modelled. The numeric value for each fraction is indicated below the corresponding segment, with a dot representing fractions <=5% The upper red bar (where present) indicates the fraction of residues that have poor fit to the electron density. The numeric value is given above the bar.

Mol	Chain	Length	Quality of chain
1	A	411	% 91% 6% •
1	B	411	% 89% 8% •

2 Entry composition (i)

There are 2 unique types of molecules in this entry. The entry contains 7000 atoms, of which 0 are hydrogens and 0 are deuteriums.

In the tables below, the ZeroOcc column contains the number of atoms modelled with zero occupancy, the AltConf column contains the number of residues with at least one atom in alternate conformation and the Trace column contains the number of residues modelled with at most 2 atoms.

- Molecule 1 is a protein called 2-amino-3-ketobutyrate coenzyme A ligase.

Mol	Chain	Residues	Atoms						ZeroOcc	AltConf	Trace
1	A	399	Total 3060	C 1913	N 556	O 579	P 1	S 11	0	0	0
1	B	399	Total 3060	C 1913	N 556	O 579	P 1	S 11	0	0	0

There are 24 discrepancies between the modelled and reference sequences:

Chain	Residue	Modelled	Actual	Comment	Reference
A	-3	MET	-	expression tag	UNP Q0K313
A	-2	GLY	-	expression tag	UNP Q0K313
A	-1	HIS	-	expression tag	UNP Q0K313
A	0	MET	-	expression tag	UNP Q0K313
A	400	LEU	-	expression tag	UNP Q0K313
A	401	GLU	-	expression tag	UNP Q0K313
A	402	HIS	-	expression tag	UNP Q0K313
A	403	HIS	-	expression tag	UNP Q0K313
A	404	HIS	-	expression tag	UNP Q0K313
A	405	HIS	-	expression tag	UNP Q0K313
A	406	HIS	-	expression tag	UNP Q0K313
A	407	HIS	-	expression tag	UNP Q0K313
B	-3	MET	-	expression tag	UNP Q0K313
B	-2	GLY	-	expression tag	UNP Q0K313
B	-1	HIS	-	expression tag	UNP Q0K313
B	0	MET	-	expression tag	UNP Q0K313
B	400	LEU	-	expression tag	UNP Q0K313
B	401	GLU	-	expression tag	UNP Q0K313
B	402	HIS	-	expression tag	UNP Q0K313
B	403	HIS	-	expression tag	UNP Q0K313
B	404	HIS	-	expression tag	UNP Q0K313
B	405	HIS	-	expression tag	UNP Q0K313
B	406	HIS	-	expression tag	UNP Q0K313
B	407	HIS	-	expression tag	UNP Q0K313

- Molecule 2 is water.

Mol	Chain	Residues	Atoms	ZeroOcc	AltConf
2	A	437	Total O<br>437 437	0	0
2	B	443	Total O<br>443 443	0	0

3 Residue-property plots (i)

These plots are drawn for all protein, RNA, DNA and oligosaccharide chains in the entry. The first graphic for a chain summarises the proportions of the various outlier classes displayed in the second graphic. The second graphic shows the sequence view annotated by issues in geometry and electron density. Residues are color-coded according to the number of geometric quality criteria for which they contain at least one outlier: green = 0, yellow = 1, orange = 2 and red = 3 or more. A red dot above a residue indicates a poor fit to the electron density (RSRZ > 2). Stretches of 2 or more consecutive residues without any outlier are shown as a green connector. Residues present in the sample, but not in the model, are shown in grey.

- Molecule 1: 2-amino-3-ketobutyrate coenzyme A ligase

Chain A:

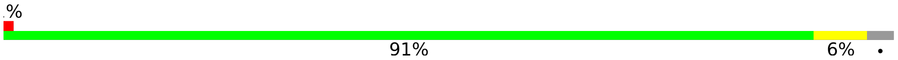

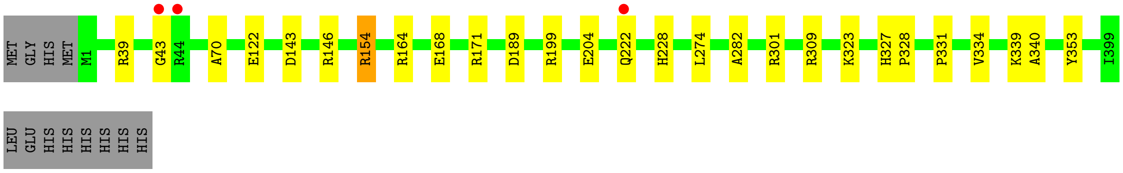

- Molecule 1: 2-amino-3-ketobutyrate coenzyme A ligase

Chain B:

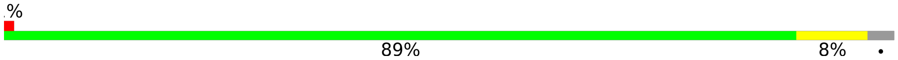

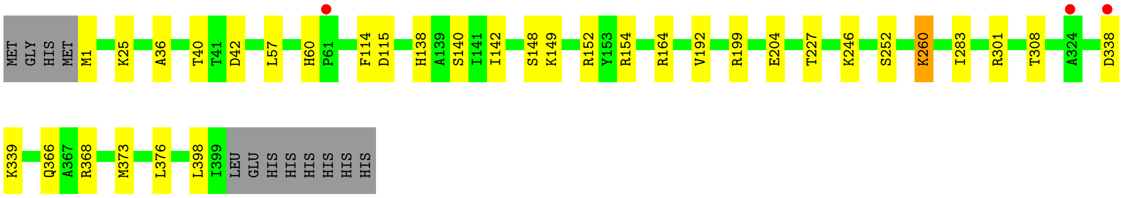

4 Data and refinement statistics (i)

Property	Value	Source
Space group	P 1 21 1	Depositor
Cell constants<br>a, b, c, α, β, γ	58.38Å 105.85Å 67.74Å<br>90.00° 105.79° 90.00°	Depositor
Resolution (Å)	41.09 – 1.80<br>45.04 – 1.80	Depositor<br>EDS
% Data completeness<br>(in resolution range)	99.9 (41.09-1.80)<br>99.9 (45.04-1.80)	Depositor<br>EDS
R_{merge}	(Not available)	Depositor
R_{sym}	(Not available)	Depositor
$< I/\sigma(I) >$ [1]	3.12 (at 1.81Å)	Xtriage
Refinement program	PHENIX 1.17.1_3660	Depositor
R, R_{free}	0.164 , 0.195<br>0.164 , 0.195	Depositor<br>DCC
R_{free} test set	3559 reflections (4.87%)	wwPDB-VP
Wilson B-factor (Å^2)	24.2	Xtriage
Anisotropy	0.078	Xtriage
Bulk solvent k_{sol}(e/Å^3), B_{sol}(Å^2)	0.36 , 45.8	EDS
L-test for twinning[2]	$< \|L\| > = 0.50$, $< L^2 > = 0.33$	Xtriage
Estimated twinning fraction	No twinning to report.	Xtriage
F_o,F_c correlation	0.96	EDS
Total number of atoms	7000	wwPDB-VP
Average B, all atoms (Å^2)	26.0	wwPDB-VP

Xtriage's analysis on translational NCS is as follows: *The largest off-origin peak in the Patterson function is 4.45% of the height of the origin peak. No significant pseudotranslation is detected.*

[1] Intensities estimated from amplitudes.

[2] Theoretical values of $< |L| >$, $< L^2 >$ for acentric reflections are 0.5, 0.333 respectively for untwinned datasets, and 0.375, 0.2 for perfectly twinned datasets.

5 Model quality (i)

5.1 Standard geometry (i)

Bond lengths and bond angles in the following residue types are not validated in this section: LLP

The Z score for a bond length (or angle) is the number of standard deviations the observed value is removed from the expected value. A bond length (or angle) with $|Z| > 5$ is considered an outlier worth inspection. RMSZ is the root-mean-square of all Z scores of the bond lengths (or angles).

Mol	Chain	Bond lengths		Bond angles	
		RMSZ	#$\|Z\|$ >5	RMSZ	#$\|Z\|$ >5
1	A	0.34	0/3082	0.56	2/4159 (0.0%)
1	B	0.35	0/3082	0.53	0/4159
All	All	0.35	0/6164	0.55	2/8318 (0.0%)

There are no bond length outliers.

All (2) bond angle outliers are listed below:

Mol	Chain	Res	Type	Atoms	Z	Observed(^{o})	Ideal(^{o})
1	A	154	ARG	NE-CZ-NH1	-7.64	116.48	120.30
1	A	154	ARG	NE-CZ-NH2	6.31	123.45	120.30

There are no chirality outliers.

There are no planarity outliers.

5.2 Too-close contacts (i)

In the following table, the Non-H and H(model) columns list the number of non-hydrogen atoms and hydrogen atoms in the chain respectively. The H(added) column lists the number of hydrogen atoms added and optimized by MolProbity. The Clashes column lists the number of clashes within the asymmetric unit, whereas Symm-Clashes lists symmetry-related clashes.

Mol	Chain	Non-H	H(model)	H(added)	Clashes	Symm-Clashes
1	A	3060	0	3034	22	0
1	B	3060	0	3034	23	0
2	A	437	0	0	7	2
2	B	443	0	0	5	2
All	All	7000	0	6068	44	2

The all-atom clashscore is defined as the number of clashes found per 1000 atoms (including

hydrogen atoms). The all-atom clashscore for this structure is 4.

All (44) close contacts within the same asymmetric unit are listed below, sorted by their clash magnitude.

Atom-1	Atom-2	Interatomic distance (Å)	Clash overlap (Å)
1:B:149:LYS:NZ	2:B:501:HOH:O	2.10	0.73
1:B:148:SER:O	2:B:502:HOH:O	2.10	0.68
1:B:338:ASP:OD1	2:B:503:HOH:O	2.14	0.64
1:B:164:ARG:NH2	1:B:204:GLU:OE2	2.30	0.64
1:A:154:ARG:HH21	1:A:154:ARG:HG2	1.64	0.61
1:A:122:GLU:HG2	2:A:829:HOH:O	2.00	0.61
1:A:154:ARG:NH2	2:A:505:HOH:O	2.33	0.61
1:A:122:GLU:HG2	2:A:584:HOH:O	2.00	0.61
1:A:309:ARG:NH2	2:A:504:HOH:O	2.31	0.60
1:A:309:ARG:NE	2:A:504:HOH:O	2.34	0.59
2:A:683:HOH:O	1:B:260:LYS:HD3	2.03	0.59
1:A:222:GLN:OE1	1:A:222:GLN:N	2.35	0.58
1:B:142:ILE:HD12	1:B:154:ARG:NH2	2.18	0.57
1:A:164:ARG:NH2	1:A:204:GLU:OE2	2.38	0.56
1:A:168:GLU:OE2	1:A:171:ARG:NH2	2.38	0.56
1:A:189:ASP:HB2	1:A:323:LYS:HD2	1.89	0.54
1:B:199:ARG:O	1:B:199:ARG:HD3	2.08	0.54
1:A:39:ARG:NH2	1:A:43:GLY:O	2.36	0.53
1:B:1:MET:N	2:B:514:HOH:O	2.42	0.51
1:A:199:ARG:O	1:A:199:ARG:HD3	2.10	0.50
1:A:323:LYS:HG3	1:A:331:PRO:HB2	1.94	0.50
1:B:152:ARG:HH11	1:B:154:ARG:NH2	2.10	0.49
1:B:138:HIS:HB2	1:B:246:LLP:H2'3	1.95	0.48
1:B:308:THR:HA	1:B:373:MET:HG3	1.95	0.48
1:B:57:LEU:HA	1:B:60:HIS:HB2	1.94	0.47
1:B:252:SER:HB3	1:B:283:ILE:HD13	1.97	0.46
1:A:274:LEU:CD2	1:B:25:LYS:HE3	2.46	0.45
1:B:114:PHE:CD2	1:B:140:SER:HB3	2.52	0.45
1:B:366:GLN:NE2	2:B:520:HOH:O	2.49	0.45
1:B:339:LYS:HG2	1:B:398:LEU:HD21	2.00	0.44
1:A:122:GLU:CG	2:A:829:HOH:O	2.64	0.43
1:B:36:ALA:HB2	1:B:376:LEU:HB3	2.00	0.43
1:B:164:ARG:HH22	1:B:204:GLU:CD	2.22	0.43
1:A:327:HIS:CG	1:A:328:PRO:HD2	2.55	0.42
1:A:334:VAL:HG21	1:A:340:ALA:HB2	2.01	0.42
1:A:154:ARG:HG2	1:A:154:ARG:NH2	2.33	0.42
1:B:40:THR:OG1	1:B:42:ASP:OD2	2.31	0.42
1:A:70:ALA:HB3	1:A:282:ALA:HA	2.02	0.41

Continued on next page...

Continued from previous page...

Atom-1	Atom-2	Interatomic distance (Å)	Clash overlap (Å)
1:A:199:ARG:HD3	1:A:199:ARG:C	2.40	0.41
1:B:366:GLN:HB3	1:B:368:ARG:HH21	1.85	0.41
1:A:143:ASP:HA	1:A:146:ARG:HD3	2.01	0.41
1:B:149:LYS:HA	1:B:149:LYS:HD3	1.82	0.41
1:B:192:VAL:HG13	1:B:227:THR:HG23	2.03	0.41
1:A:164:ARG:HH22	1:A:204:GLU:CD	2.22	0.40

All (2) symmetry-related close contacts are listed below. The label for Atom-2 includes the symmetry operator and encoded unit-cell translations to be applied.

Atom-1	Atom-2	Interatomic distance (Å)	Clash overlap (Å)
2:A:762:HOH:O	2:B:514:HOH:O[1_455]	1.55	0.65
2:A:508:HOH:O	2:B:514:HOH:O[1_455]	1.78	0.42

5.3 Torsion angles (i)

5.3.1 Protein backbone (i)

In the following table, the Percentiles column shows the percent Ramachandran outliers of the chain as a percentile score with respect to all X-ray entries followed by that with respect to entries of similar resolution.

The Analysed column shows the number of residues for which the backbone conformation was analysed, and the total number of residues.

Mol	Chain	Analysed	Favoured	Allowed	Outliers	Percentiles	
1	A	396/411 (96%)	383 (97%)	13 (3%)	0	100	100
1	B	396/411 (96%)	385 (97%)	11 (3%)	0	100	100
All	All	792/822 (96%)	768 (97%)	24 (3%)	0	100	100

There are no Ramachandran outliers to report.

5.3.2 Protein sidechains (i)

In the following table, the Percentiles column shows the percent sidechain outliers of the chain as a percentile score with respect to all X-ray entries followed by that with respect to entries of similar resolution.

The Analysed column shows the number of residues for which the sidechain conformation was

analysed, and the total number of residues.

Mol	Chain	Analysed	Rotameric	Outliers	Percentiles	
1	A	307/318 (96%)	303 (99%)	4 (1%)	69	62
1	B	307/318 (96%)	304 (99%)	3 (1%)	76	71
All	All	614/636 (96%)	607 (99%)	7 (1%)	73	68

All (7) residues with a non-rotameric sidechain are listed below:

Mol	Chain	Res	Type
1	A	228	HIS
1	A	301	ARG
1	A	339	LYS
1	A	353	TYR
1	B	115	ASP
1	B	260	LYS
1	B	301	ARG

Sometimes sidechains can be flipped to improve hydrogen bonding and reduce clashes. All (1) such sidechains are listed below:

Mol	Chain	Res	Type
1	B	366	GLN

5.3.3 RNA (i)

There are no RNA molecules in this entry.

5.4 Non-standard residues in protein, DNA, RNA chains (i)

2 non-standard protein/DNA/RNA residues are modelled in this entry.

In the following table, the Counts columns list the number of bonds (or angles) for which Mogul statistics could be retrieved, the number of bonds (or angles) that are observed in the model and the number of bonds (or angles) that are defined in the Chemical Component Dictionary. The Link column lists molecule types, if any, to which the group is linked. The Z score for a bond length (or angle) is the number of standard deviations the observed value is removed from the expected value. A bond length (or angle) with $|Z| > 2$ is considered an outlier worth inspection. RMSZ is the root-mean-square of all Z scores of the bond lengths (or angles).

Mol	Type	Chain	Res	Link	Bond lengths			Bond angles		
					Counts	RMSZ	$\#\|Z\| > 2$	Counts	RMSZ	$\#\|Z\| > 2$
1	LLP	A	246	1	23,24,25	0.54	0	25,32,34	0.74	0

Mol	Type	Chain	Res	Link	Bond lengths			Bond angles		
					Counts	RMSZ	#|Z| > 2	Counts	RMSZ	#|Z| > 2
1	LLP	B	246	1	23,24,25	0.46	0	25,32,34	0.71	0

In the following table, the Chirals column lists the number of chiral outliers, the number of chiral centers analysed, the number of these observed in the model and the number defined in the Chemical Component Dictionary. Similar counts are reported in the Torsion and Rings columns. '-' means no outliers of that kind were identified.

Mol	Type	Chain	Res	Link	Chirals	Torsions	Rings
1	LLP	A	246	1	-	5/16/17/19	0/1/1/1
1	LLP	B	246	1	-	3/16/17/19	0/1/1/1

There are no bond length outliers.

There are no bond angle outliers.

There are no chirality outliers.

All (8) torsion outliers are listed below:

Mol	Chain	Res	Type	Atoms
1	A	246	LLP	C5'-OP4-P-OP1
1	A	246	LLP	O-C-CA-CB
1	B	246	LLP	O-C-CA-CB
1	A	246	LLP	C4-C4'-NZ-CE
1	B	246	LLP	CG-CD-CE-NZ
1	B	246	LLP	C4-C4'-NZ-CE
1	A	246	LLP	C5'-OP4-P-OP3
1	A	246	LLP	CG-CD-CE-NZ

There are no ring outliers.

1 monomer is involved in 1 short contact:

Mol	Chain	Res	Type	Clashes	Symm-Clashes
1	B	246	LLP	1	0

5.5 Carbohydrates (i)

There are no monosaccharides in this entry.

5.6 Ligand geometry (i)

There are no ligands in this entry.

5.7 Other polymers (i)

There are no such residues in this entry.

5.8 Polymer linkage issues (i)

There are no chain breaks in this entry.

6 Fit of model and data (i)

6.1 Protein, DNA and RNA chains (i)

In the following table, the column labelled '#RSRZ> 2' contains the number (and percentage) of RSRZ outliers, followed by percent RSRZ outliers for the chain as percentile scores relative to all X-ray entries and entries of similar resolution. The OWAB column contains the minimum, median, 95th percentile and maximum values of the occupancy-weighted average B-factor per residue. The column labelled 'Q< 0.9' lists the number of (and percentage) of residues with an average occupancy less than 0.9.

Mol	Chain	Analysed	<RSRZ>	#RSRZ>2			OWAB(Å^2)	Q<0.9
1	A	398/411 (96%)	-0.41	3 (0%)	86	84	17, 24, 37, 48	0
1	B	398/411 (96%)	-0.30	3 (0%)	86	84	17, 24, 38, 44	0
All	All	796/822 (96%)	-0.36	6 (0%)	86	84	17, 24, 38, 48	0

All (6) RSRZ outliers are listed below:

Mol	Chain	Res	Type	RSRZ
1	B	338	ASP	3.0
1	A	44	ARG	2.5
1	A	222	GLN	2.4
1	A	43	GLY	2.2
1	B	61	PRO	2.1
1	B	324	ALA	2.1

6.2 Non-standard residues in protein, DNA, RNA chains (i)

In the following table, the Atoms column lists the number of modelled atoms in the group and the number defined in the chemical component dictionary. The B-factors column lists the minimum, median, 95th percentile and maximum values of B factors of atoms in the group. The column labelled 'Q< 0.9' lists the number of atoms with occupancy less than 0.9.

Mol	Type	Chain	Res	Atoms	RSCC	RSR	B-factors(Å^2)	Q<0.9
1	LLP	A	246	24/25	0.96	0.09	17,20,23,24	0
1	LLP	B	246	24/25	0.97	0.09	18,20,22,23	0

6.3 Carbohydrates (i)

There are no monosaccharides in this entry.

6.4 Ligands (i)

There are no ligands in this entry.

6.5 Other polymers (i)

There are no such residues in this entry.
