# Supplementary material for: Chemoenzymatic synthesis of 3-ethyl-2,5-dimethylpyrazine by L-threonine 3-dehydrogenase and 2-amino-3-ketobutyrate CoA ligase/L-threonine aldolase
Source: Commun Chem. 2021 Jul 16;4:108. doi: 10.1038/s42004-021-00545-8 (PMC9814548; doi:10.1038/s42004-021-00545-8)
Supplement: Supplementary file 10 — Description of Additional Supplementary Files [file 42004_2021_545_MOESM10_ESM.pdf]

Description of Additional Supplementary Files

File Name: Supplementary Data 1
Description: Protein sequences of CnTDH and CnKBL

File Name: Supplementary Data 2
Description: Nucleotide sequences of CnTDH and CnKBL

File Name: Supplementary Data 3
Description: Validation report of 7BXP

File Name: Supplementary Data 4
Description: Validation report of 7BXQ

File Name: Supplementary Data 5
Description: Validation report of 7BXR

File Name: Supplementary Data 6
Description: Validation report of 7BXS
